# Uptake of Isoniazid Preventive Therapy among Under-Five Children: TB Contact Investigation as an Entry Point

**DOI:** 10.1371/journal.pone.0155525

**Published:** 2016-05-19

**Authors:** Yared Tadesse, Nigussie Gebre, Shallo Daba, Zewdu Gashu, Dereje Habte, Nebiyu Hiruy, Solomon Negash, Kassahun Melkieneh, Degu Jerene, Yared K. Haile, Yewulsew Kassie, Muluken Melese, Pedro G. Suarez

**Affiliations:** 1 Management Sciences for Health, Help Ethiopia Address the Low Performance of Tuberculosis (HEAL TB) Project, Addis Ababa, Ethiopia; 2 Amhara Regional Health Bureau, Bahir Dar, Ethiopia; 3 Oromia Regional Health Bureau, Addis Ababa, Ethiopia; 4 United States Agency for International Development (USAID), Addis Ababa, Ethiopia; 5 Management Sciences for Health, Health Programs Group, Arlington, Virginia, United States of America; University of Cape Town, SOUTH AFRICA

## Abstract

A child’s risk of developing tuberculosis (TB) can be reduced by nearly 60% with administration of 6 months course of isoniazid preventive therapy (IPT). However, uptake of IPT by national TB programs is low, and IPT delivery is a challenge in many resource-limited high TB-burden settings. Routinely collected program data was analyzed to determine the coverage and outcome of implementation of IPT for eligible under-five year old children in 28 health facilities in two regions of Ethiopia. A total of 504 index smear-positive pulmonary TB (SS+) cases were reported between October 2013 and June 2014 in the 28 health facilities. There were 282 under-five children registered as household contacts of these SS+ TB index cases, accounting for 17.9% of all household contacts. Of these, 237 (84%) were screened for TB symptoms, and presumptive TB was identified in 16 (6.8%) children. TB was confirmed in 5 children, producing an overall yield of 2.11% (95% confidence interval, 0.76–4.08%). Of 221 children eligible for IPT, 64.3% (142) received IPT, 80.3% (114) of whom successfully completed six months of therapy. No child developed active TB while on IPT. Contact screening is a good entry point for delivery of IPT to at risk children and should be routine practice as recommended by the WHO despite the implementation challenges.

## Introduction

Tuberculosis (TB) remains one of the world’s deadliest communicable diseases. In 2014 globally 9.6 million people are estimated to have fallen ill with TB, amongst which children constituted 1.0 million of the total. The actual burden is likely to be higher, because diagnosing TB in children is challenging and is a low priority in low-resource settings [1].

Ethiopia is also one of the 22 high-burden countries for TB, and childhood TB accounts for 13% of the overall TB burden with case notification among children below 15 years of age estimated to be 15,917 [1]. Even this could be an underestimate due to difficulty in confirmation of diagnosis of TB in children. The World Health Organization (WHO) states in its post-2015 global recommendation the need for preventive treatment of persons at high risk as one strategy for prevention, care, and control of TB [2]. There is a road map for childhood TB and global pediatric TB guidelines and preventive therapy is one of the key interventions. Ethiopia has also developed a national roadmap for prevention and control of childhood TB which emphasizes on the implementation of contact screening & provision of isoniazid preventive therapy (IPT) for under-5 years as one intervention to prevent childhood TB. However, the gap is in the implementation of the recommended strategies/activities.

Isoniazid (INH) preventive therapy (IPT) is currently recommended for the treatment of latent TB infection among people living with HIV and children under five years of age who are contacts of patients with TB [3]. Isoniazid prophylaxis can reduce the risk of developing tuberculosis by 59% among children aged 15 years or Younger [4]. The WHO also recommends offering IPT for at least six months to all children below five years of age who have household contact with an infectious TB case, after ruling out active TB disease [5]. Ethiopia has accepted and is implementing the WHO’s recommendation of a six-month course of IPT for under-five children who have a history of contact with a sputum-smear-positive (SS+) pulmonary TB index patient, after ruling out the presence of active TB disease [6].

Even though IPT is a global recommendation, its initiation and completion rate is sub-optimal. The level of awareness among health care providers, interruption of INH supply, co-infection with HIV, lack of recording tools for IPT and distance from health facilities affect uptake of the service in different settings. The IPT initiation and completion rates reported in research settings ranged between 18–33% and 23–50% respectively [7–10]. Whereas the IPT initiation and completion rates reported in program implementation settings were between 21–58% and 13% respectively [11–12].

Most studies conducted on IPT focus on the setting of TB/HIV co-infected populations and research settings. However, research settings on IPT uptake may be more controlled as compared to routine implementation setting which reflect real life experiences and bottlenecks. We present the IPT implementation experience under routine program intervention; regional and health facility type comparisons were also made to understand the experience of IPT implementation in diverse settings. Hence the objective of this implementation study was to assess the effectiveness of contact screening as an entry point for IPT implementation and treatment among eligible under-five children initiated under normal program conditions

## Methods

### Ethics

Ethical approval was obtained from the Amhara and Oromia Regional Health Bureau institutional review boards (IRBs). Patients’ identifier information was anonymized and de-identified prior to analysis. The finding of the analysis will be shared with federal ministry of health and regional health bureaus for evidence based decision making.

### Setting

We implemented household contact screening and identified eligible children for IPT implementation in a regular program setting in the Amhara and Oromia Regional States of the Federal Democratic Republic of Ethiopia, with case notification rate (CNR) of 117 and 146 per 100,000 respectively [13]. In the two regions, there are 64 hospitals and 2,122 health centers providing TB services. The Help Ethiopia Address the Low TB Performance (HEAL-TB) Project, funded by the US Agency for International Development and operated in collaboration with the Amhara and Oromia Regional Health Bureaus, standardized the activity of contact screening of SS+ pulmonary TB index cases. Contact screening of SS+ pulmonary TB cases was used as entry point to identify and enroll eligible under-five contacts in IPT. Health workers were oriented on the importance of IPT through individualized mentorship and continuing medical education sessions specifically designed for mid- and low-level health workers. Additionally, job aids and recording and reporting formats were supplied for routine use. We previously reported our experience in contact investigation and its yield [14].

### Study Population and Data Source

**Fig 1:**
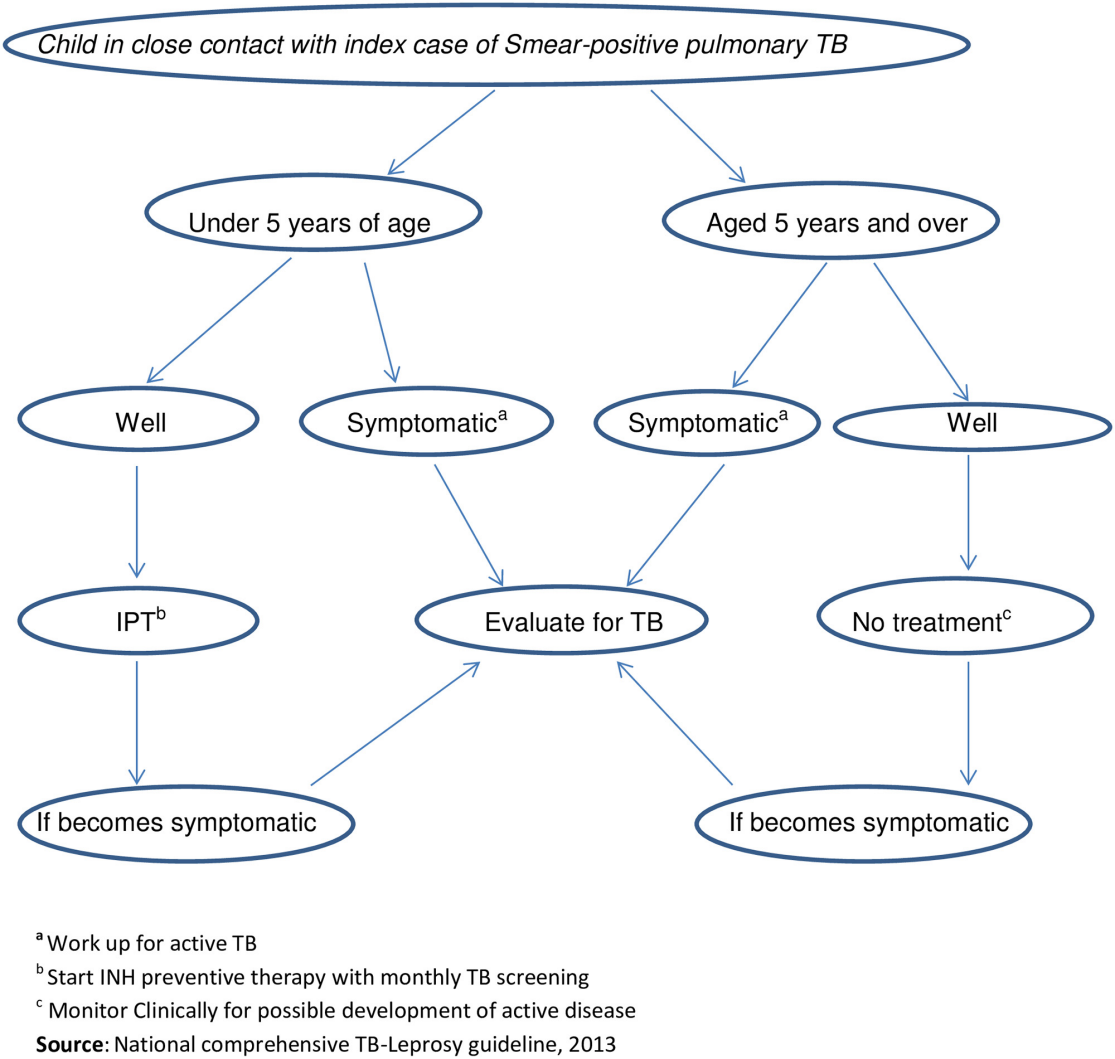
Algorithm for childhood TB management according to the national guidelines in Ethiopia.

Contact screening of family members of index SS+ pulmonary TB patients is routinely conducted by TB focal persons at TB clinics, while index TB patients receive DOTS at TB clinics. We used the national clinical TB screening algorithm (Fig 1). Eligible under-five children for IPT are initiated and followed in the TB clinic where monthly refill, symptom screening and care taker counselling, was performed as per the national TB/Leprosy guideline [6]. We used the data routinely recorded in the contact investigation register at health facilities providing TB program services. Data was retrieved from health facilities every quarter. Based on our routine program data, we analyzed IPT-related information from 28 health facilities (7 hospitals and 21 health centers) out of the total 64 hospitals & 2,122 health centers in the period between October 2013 and June 2014. The 28 health facilities were selected based on their high TB case load and also in that we were able to re-count the routinely submitted IPT report by zonal TB focal persons in these health facilities. Additionally we made sure that the selection covered different geographic areas with different settings. We were able to do IPT data quality checking in all 28 health facilities.

Data was gathered on the following variables: number of SS+ cases; number of household contacts; proportion of under-five-year-old household contacts for whom symptom-based screening was done as per the national recommendation (Fig 1); and the number of eligible under-five children who were started on and completed IPT.

### Data Analysis

Data was entered in Excel and exported to Stata for statistical analysis. We computed frequency, percentage, and 95% confidence interval to present the findings. We used the chi-square test to assess differences between categories. P-values less than 0.05 were considered statistically significant.

## Results

**Fig 2:**
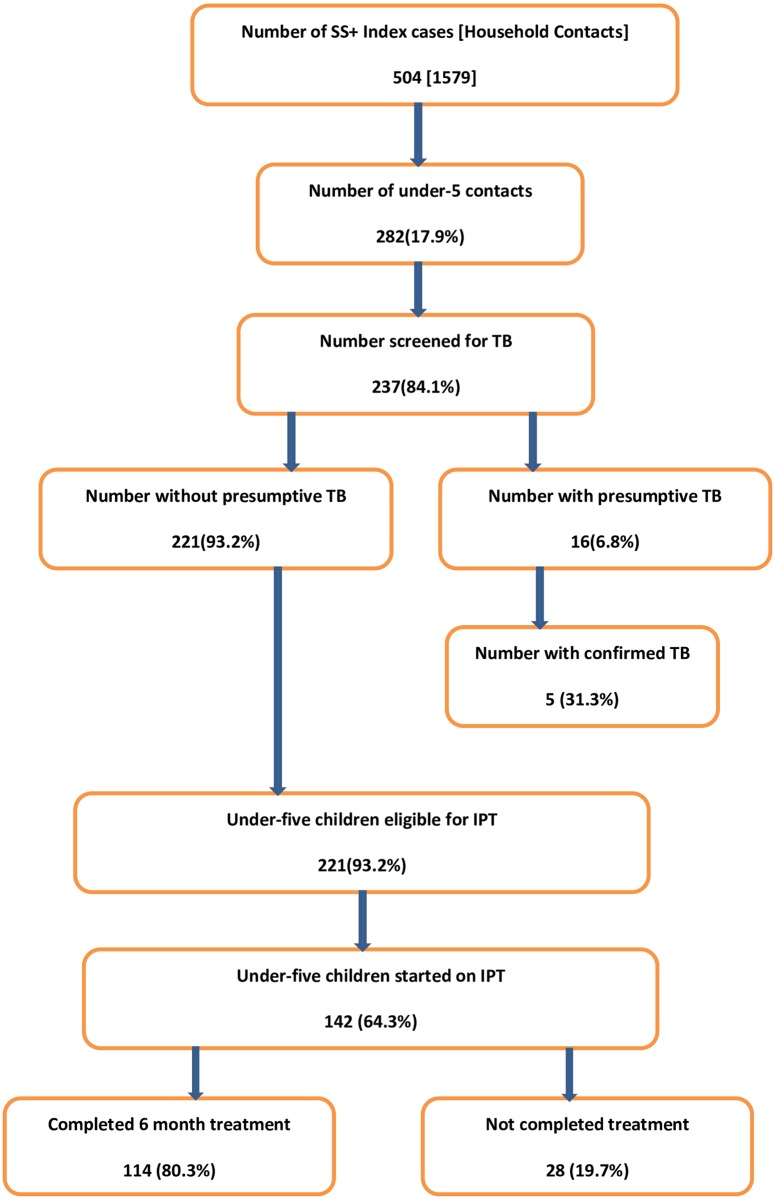
Contact screening and IPT among under-five children, Oct. 2013-June 2014, Ethiopia.

**Fig 3:**
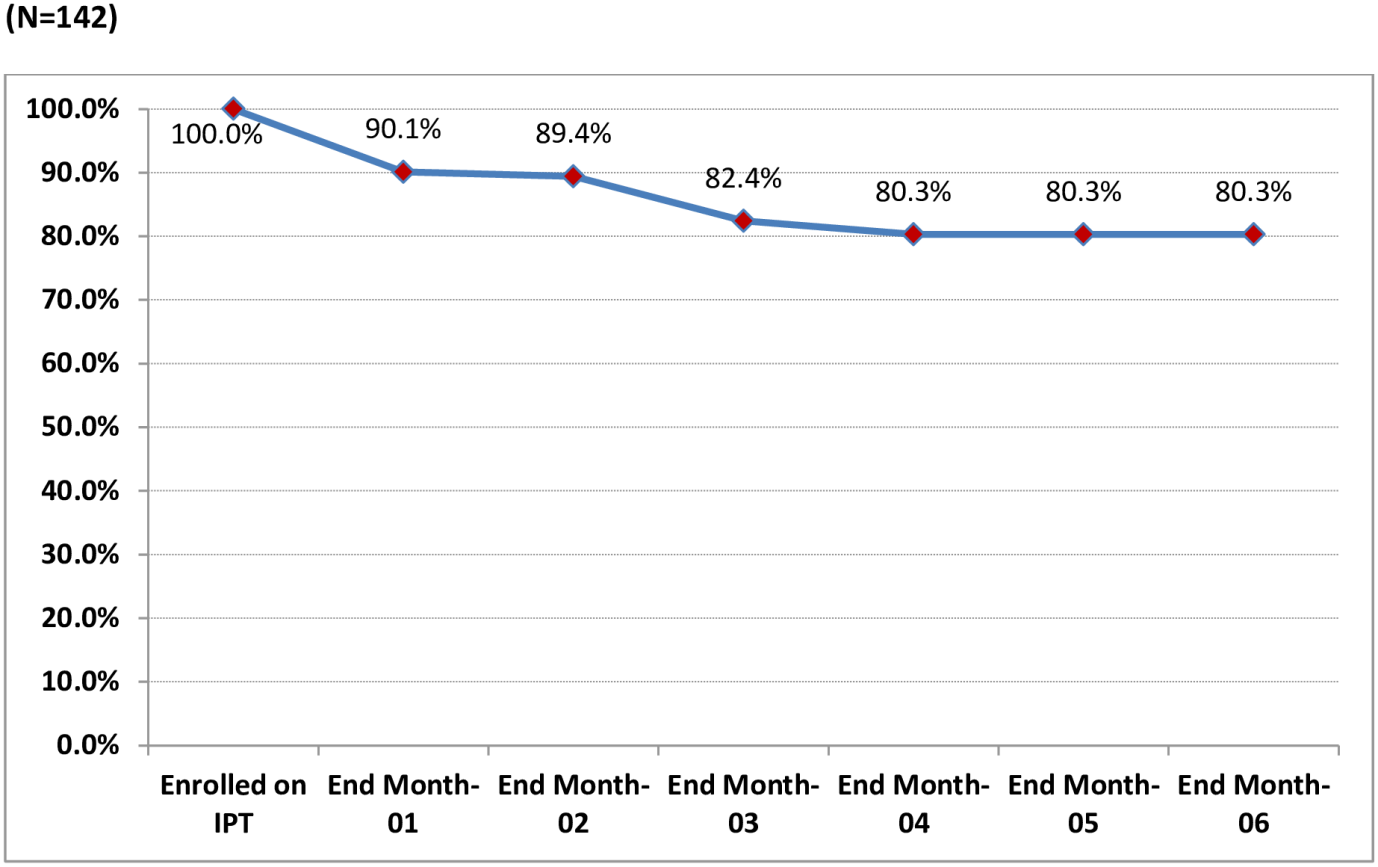
Percentage of under-five children retained on IPT during six-month follow-up.

A total of 504 index SS+ cases were reported between October 2013 and June 2014. There were 282 under-five children registered as household contacts of the SS+ index cases, accounting for 17.9% of all household contacts (Fig 2). Of these, 237 (84%) were screened for TB using the national symptom-based TB screening algorithm [6] and 16(6.8%) were identified as having presumptive TB (Fig 1). TB was confirmed in 5 children, producing a yield of 2.11% (95% confidence interval, 0.76–4.08%). Of 221 children without presumptive TB and eligible for IPT, 142 (64.3%) received IPT, of whom 114 (80.3%) completed the six-month course while 28 (19.7%) interrupted treatment (Fig 3). Among the children who interrupted IPT treatment (n = 28), 14 children did so in the first month, 1 child in the second month, 10 children in the third month, and 3 in the fourth.

Of the total of 852 household contacts in Oromia Region, 180 (21%) were under-five child contacts, while in Amhara under-five children constituted 102 (36%) of the total 727 household contacts. There was no regional variation in terms of the proportion of the presumptive TB identified, active TB detected, IPT coverage, and completion rate of IPT treatment among the children (p > 0.05).

The proportion of registered under-five child household contacts who were clinically screened for TB at the health centers and hospitals was 85% and 82%, respectively (p = 0.26). Health centers contributed more than 70% (157/221) of the IPT-eligible under-five children and 62% (10/16) of the presumptive TB cases identified. Hospitals contributed nearly 30% (64/221) of IPT-eligible children and 38% (6/16) children with presumptive TB. The proportion of the eligible children put on IPT at health centers was 65% (102/157), while it was 62.5% (40/64) at hospitals. The IPT completion rate was 85% (80/102) at health centers as compared to 78.4% (34/40) at hospitals, but the difference was not statistically significant (p > 0.05) (Table 1).

**Table 1:** Contact Screening and IPT among Under-five Children, by Health Facility Type.

Variables	Facility Type		
	Health center	Hospital	P- value ^a^
Number of SS+ index cases	334	170	
Number of total household contacts	1036	543	
Ratio of index cases to household contacts	1:3	1:3	
Number (%) of under-five contacts	197 (19%)	85 (15.7%)	0.19
Number (%) of under-fives screened for TB	167 (85%)	70 (82%)	0.26
Number (%) of under-fives with presumptive TB	10 (6%)	6 (8.6%)	0.23
Number of under-fives diagnosed with TB and treated	2	3	
Number of children eligible for IPT	157	64	
Number (%) of children put on IPT	102 (64.9%)	40 (62.5%)	0.70
Number (%) of children who completed the six-month IPT	80 (78.4%)	34 (85%)	0.08

SS+, sputum-smear positive; IPT, isoniazid preventive therapy.

^a^ Two-sample test for proportions using Stata.

## Discussion

This study demonstrated the feasibility of providing a six-month course of IPT under routine program conditions to eligible under-five children who were in close contact with SS+ index cases. We confirmed that contact investigation was an important entry point to identify under-five children with TB and those who needed preventive therapy against TB. The IPT completion rate was within a reasonable range, but the factors contributing to IPT interruption such as lack of leadership by national TB control programs (NTPs) of preventive interventions such as IPT, low awareness & experience of health care workers of the benefits of IPT, providers’ perceived fear of toxicity of INH & generating drug resistance, lack of parent/caretaker knowledge as to benefits of IPT [15, 16] need to be addressed.

The WHO recommends clinical evaluation of household contacts of SS+ index cases for active TB. The two main purposes of contact screening and management are: first, to identify contacts of all ages with undiagnosed TB disease among the contacts of an index case, and second, to provide preventive therapy for contacts without TB disease who are susceptible to developing disease following recent infection [5].

The IPT initiation rate of 64.3% in our study is slightly lower than the 68% reported from South Africa [17]. However, it is higher than the 33% rate in southern India [12] and the 18% reported in Timor-Leste [9]. In addition, a study in Malawi showed that only 23 (6%) of 365 under-five child household contacts received IPT [18].

Even though the setting for IPT implementation among HIV infected populations is different from childhood IPT, the IPT initiation rate in the current study is much better than the 39% reported among a cohort of HIV-infected people in Southern Ethiopia [19], and 3.8% reported from Addis Ababa [20]. Uptake and adherence to IPT among HIV-infected people was difficult due to the use of multiple drugs at a time which is not the case for IPT among children [14, 15, 19].

In our study, over two-thirds of eligible children received IPT, with most of them successfully completing the recommended dose. The IPT completion rate of 80% in this study was much higher than the 23% reported from southern India, 24% reported among HIV-infected patients [18] and 12% reported from another study in southern Ethiopia [21. In Pakistan, of 184 under-five children enrolled in IPT, 60 (32.6%) completed six months of IPT [22]. But in the South African report [18], only 15% achieved four months of therapy. Hence, the higher IPT completion rate in our study is encouraging, but more effort is needed to ensure 100% adherence.

Achievement of a higher IPT completion rate in this study also demonstrates that IPT is feasible in a resource-limited setting and that contact investigation of index TB cases can be used as a core entry point for TB case detection and prevention in the childhood population in similar settings. With further strengthening of health workers’ capacity, even higher rates of initiation and completion are within reach [23]

Of the total of 28 IPT interrupters, 25 (89%) children discontinued within the first three months after initiation of IPT. There was no interruption after completion of the fourth month of preventive treatment. The major reason for the high interruption rate in a study done in southern Ethiopia was families’ refusal to have an otherwise healthy child treated for six months in a TB clinic (where TB treatment is provided) [21], similarly the long duration of treatment was a factor in 28% of cases in India [12]. Although 28 children interrupted preventive therapy in this study, evidence has shown that IPT is safe and well tolerated by children; major potential serious adverse events, including hepatotoxicity and pyridoxine deficiency, are rare in children [24–26]. As this was a routine reported data, there was no specific side effect related information. In a Kenyan study, among HIV-infected children below 14 years of age who were started on IPT, the main reasons for discontinuation of preventive therapy were developing active TB, frequent treatment interruptions, and being lost to follow-up [27].

It is encouraging to attain an overall IPT completion rate of 80%, but we still need to understand the factors contributing to IPT interruption early in the course of therapy and to address the remaining 20% who interrupted IPT. Further studies are needed to provide evidence to improve completion rate and monitor adherence of IPT. Since IPT completion rather than initiation is the key protective indicator, studies on factors that contribute to completion of unsupervised IPT, such as parent/caretaker education, uninterrupted drug supply, and tracing of those lost to follow-up, should be emphasized. The effectiveness of delivering IPT in kits and directly observing parent-child pairs should be evaluated as there exists evidence showing that introduction of individualized TB treatment kit has beneficial effect in ensuring uninterrupted drug supply with fewer stock outs, minimizing lost to follow ups and building patient confidence with improved adherence to TB treatment [28].

Screening of contacts of TB cases helps to identify at-risk contacts, such as HIV-infected under-fives who require preventive therapy, and of any age who have active TB [29]. Contact screening also contributes significantly to identify children with active TB disease early to prevent childhood morbidity and mortality. In one study it was found that there is an eight-fold increased risk of TB mortality in children living in households with someone who has active TB [30]. Moreover, about 81% of missed opportunities for IPT in at-risk children who later presented with confirmed TB were under three years of age, 25% had disseminated TB, and 5% died [31]. Yet our study demonstrated that 45 (16%) children of index TB cases were not screened for TB. Another study in Malawi in 2006 reported that only 8% of parents with SS+ TB brought their children to a clinic for screening despite provision of clear information [32]. In Addis Ababa, only 23.6% of index cases reported that a health care worker instructed them to bring their child for TB screening [19]. These gaps could indicate that health care providers should also be equipped with the knowledge, skills, and tools to counsel parents or caregivers about the importance of screening children who are in contact with TB patients and about preventive treatment even for otherwise healthy children.

In terms of the capacity to initiate IPT for eligible children, 65% of eligible children at health centers were initiated on IPT, while 62.5% of eligible children at hospitals were initiated on IPT, which is not a statistically significant difference (P > 0.05). This indicates that mid-level health workers at peripheral health facilities can implement IPT and that IPT can be decentralized in order to make it more accessible to rural communities. The success can be attributed to capacity building of health care workers, especially at the primary health care level through training, mentorship, program monitoring, and supportive supervision. As reported elsewhere [33], provision of job aids (screening algorithms) and monitoring tools provided by the project were instrumental in improving the awareness of program managers and health care providers in implementing IPT as a childhood TB prevention strategy.

A review of clinical trials indicated that IPT reduces the risk of TB by about 60% among the infected contacts of all ages [34] and that the efficacy of IPT is even higher in children, at 80–90% [35]. The review also showed that 1 case of active TB (over the next five years) can be prevented for every 35 TB contacts who are prescribed INH for six months [35]. In the year of data collection, there were 38,403 registered cases of SS+ TB in the two regions support by the HEAL-TB Project (unpublished report). Extrapolating similar ratios of under-five contacts per index case and IPT completion rate in this analysis to the project regions, there would be 21,487 under-five contacts, of whom 8,686 had completed IPT. Accordingly, contact investigation and IPT intervention for the under-fives in the two largest regions of Ethiopia likely prevented about 248 cases of TB-related morbidity in under-five children (1 in 35 treated with IPT), provided that the findings in this analysis represent the overall project. If IPT had reached all under-five contacts without presumptive TB in the year, the corresponding number of children prevented from acquiring active TB in the two regions would have been 572.

The study has some limitations. Because we carried out the analysis in purposively selected health facilities, its findings might not be generalizable to the remaining health facilities. Also unavailability of detailed data with respect to gender, age and smear positivity grading of the index case can be considered as limitations. However, the findings provided program-level evidence about the actual implementation of contact screening and IPT. Because contact screening and provision of IPT form part of regular program implementation, the development of TB among those who completed IPT was not assessed.

## Conclusions

The findings demonstrated that tracing infants and young children who are contacts of infectious TB cases and offering them preventive therapy was feasible in the regular DOTS program setting. Services for IPT at health centers and at hospitals showed comparable IPT initiation and completion rates. Contact screening is a feasible entry point for IPT and the IPT completion rate was good, but the remaining gaps should be addressed. Comprehensive support provided by the project was instrumental in improving the awareness of program managers and health care providers in implementing IPT as a childhood TB prevention strategy.

Further studies are needed to better understand factors contributing to IPT interruption early in the course of therapy, the feasibility of delivering IPT in kit form, and the long-term benefits of IPT in terms of reducing TB-related morbidity and mortality among under-five child contacts of SS+ TB cases.

## Supporting Information

S1 Table List of Health Facilities. (DOCX)

S1 Text Ethical Approval from Amhara Regional Health Bureau. (JPG)

S2 Text Ethical Approval from Oromia Regional Health Bureau. (PDF)
