# Supplementary material for: Uptake of Isoniazid Preventive Therapy among Under-Five Children: TB Contact Investigation as an Entry Point
Source: PLoS One. 2016 May 19;11(5):e0155525. doi: 10.1371/journal.pone.0155525 (PMC4873181; doi:10.1371/journal.pone.0155525)
Supplement: S1 Table — (DOCX) [file pone.0155525.s001.docx]

**List of Health Facilities utilized for data collection**

**Amhara Region**

1. awi/Afesa Health Center (HC)
2. Enewari HC
3. D/Berhan Hospital
4. Meragna HC
5. Enat Hospital
6. Deneba HC
7. Chacha HC
8. Maksegnit HC
9. Shewarobit HC
10. Artumafursi(Chefarobit) HC
11. TachGayint(ArbGebeya) HC
12. Woreta HC

**Oromia Region**

1. Negele Hospital
2. Kara Gora HC
3. Gori HC
4. Enango HC
5. Chole HC
6. Gonde HC
7. Chalbesa HC
8. Tore HC
9. Metari HC
10. Moyale Hospital
11. Bulehora Hospital
12. Kuni HC
13. Chiro Hospital
14. Gelemso Hospital
15. Sekina HC
16. Micheta HC
