# Supplementary figures and images for: Uptake of Isoniazid Preventive Therapy among Under-Five Children: TB Contact Investigation as an Entry Point
Source: PLoS One. 2016 May 19;11(5):e0155525. doi: 10.1371/journal.pone.0155525 (PMC4873181; doi:10.1371/journal.pone.0155525)

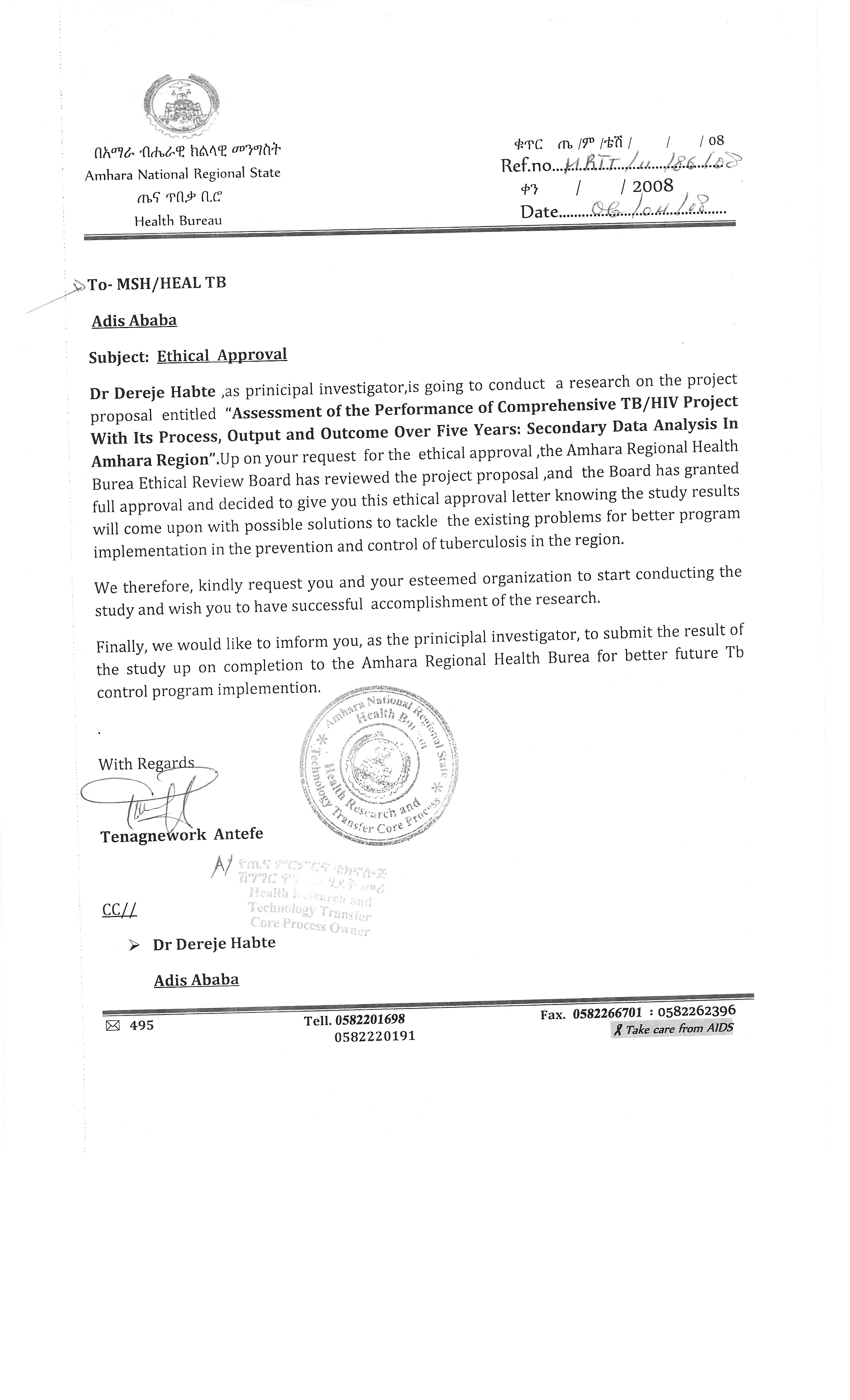

Supplement: S1 Text — (JPG) [file pone.0155525.s002.jpg]
