# Supplementary material for: Uptake of Isoniazid Preventive Therapy among Under-Five Children: TB Contact Investigation as an Entry Point
Source: PLoS One. 2016 May 19;11(5):e0155525. doi: 10.1371/journal.pone.0155525 (PMC4873181; doi:10.1371/journal.pone.0155525)
Supplement: S2 Text — (PDF) [file pone.0155525.s003.pdf]

IIROO EEGUMSA FAYYAA OROMIYAA

OROMIA HEALTH BUREAU

የኦሮሚያ ጤና ጥበቃ ቢሮ

Lakk/Ref. No. BEFOAHBTHQQ/10746-8

Guyyaa /Date 12/08/08

To MSH –HEAL TB

Addis Ababa

Subject: Ethical approval

Dr. Dereje Hante is going to conduct research in our Region on the project proposal titled" **Assessment of the performance of comprehensive TB/HIV Project with Its Process, Output and Outcome over the Five Years: Secondary Data Analysis in Oromia, Region.** Based on the principal investigator's request for ethical approval, the Oromia Regional Health Bureau research ethics review board has reviewed the aforementioned project protocol in an expedited manner. We are writing to advise you that Oromia Regional Health Bureau ethical review board has granted full approval and decided to give this ethical approval letter internalizing the existing problem and the study eventually come up with possible solution that will help for the control and prevention of Tuberculosis in the future.

We, therefore, request you as PI and your esteemed organization to ensure the commencement and conduct of the study accordingly and wish for the successful completion of the study. Finally, we would like to inform PI to submit the result of the study up on completion to ORHB for implementation purpose.

With best regards

Amanuel Yadassa

Signature Tel-0910248162

Name of PI: "**Dr. Dereje Habte**"

CC

Dr. Dereje Habte

Addis Ababa

Address: Tel: +251-11-371-72-77, Fax: +251-11-371-72-27 P.O.Box.24341 E-mail: ohbhead@telecom.net.et
ADDIS ABABA/FINFINNE-ETHIOPIA
